# 

**Published:** 1888-04

**Authors:** 


					﻿Vol. IX.
APRIL, 1888.
No. 4.
THZ
imiitn prae 1 lim
MONTHLY dOTONAI
DEVOTED TO
DENTAL AND ORAL SCIENCE.
W. C. BARRETT, M. D„ D. D. S.;
EDITOR.
The Hew York Brutal Journal Association,
PUBLISHERS,
No. 35 West 46th Street, New York
AND
■ No. 208 Franklin St., BnfFalo, N.Y.
$2.50 Per Annum in Advance, .... Single Copies, 25 Cents
Entered at the Post-Office, Buffalo, N. Y., as Second-Class matter.
				

